# Physical exercise as a non-pharmacological strategy to enhance glymphatic function

**DOI:** 10.1016/j.ibneur.2026.01.014

**Published:** 2026-01-30

**Authors:** Arman Ghayourvahdat, Hannaneh Azimizonuzi, Moslem Ahmed

**Affiliations:** a Inventor Member, Iran Representative Office of the International Federation of Inventors’ Associations (IFIA), Tehran, Iran; b Department of Neurology, Tehran University of Medical Sciences, Tehran, Iran

**Keywords:** Glymphatic System, Physical Exercise, Cerebrospinal Fluid Dynamics, Aquaporin-4, Brain Clearance, Neurodegeneration

## Abstract

The glymphatic system plays a critical role in clearing metabolic waste and neurotoxic proteins from the brain, and its dysfunction is implicated in neurodegenerative diseases such as Alzheimer’s disease (AD). Emerging evidence indicates that physical exercise enhances glymphatic function through multiple mechanisms, including increased cerebrospinal fluid (CSF) influx, improved perivascular clearance, astrocytic aquaporin-4 (AQP4) polarization, and modulation of vascular and sleep-dependent processes. Preclinical studies demonstrated that voluntary wheel running and aerobic exercise reduce amyloid-β (Aβ) accumulation, attenuate neuroinflammation, and improve cognitive performance in both aging and AD mouse models, with benefits being highly dependent on AQP4 expression and the timing of intervention. Translational evidence in humans showed that structured aerobic and multicomponent exercise increases glymphatic and meningeal lymphatic activity, enhances vascular dynamics, reduces systemic inflammation, and improves sleep quality, leading to measurable cognitive gains. Despite these promising findings, methodological challenges—such as limitations of non-invasive imaging, difficulty establishing causality, and reliance on short-term interventions—highlight the need for longitudinal, multimodal studies that integrate imaging, cardiovascular, sleep, and cognitive metrics. Collectively, these data suggest that exercise represents a potent non-pharmacological strategy to augment glymphatic clearance, preserve neural homeostasis, and reduce the risk of cognitive decline. This review will summarize evidence on exercise-induced glymphatic enhancement, highlight mechanisms, and identify research gaps for future studies on brain health.

## Graphical Abstract

**Figure:**
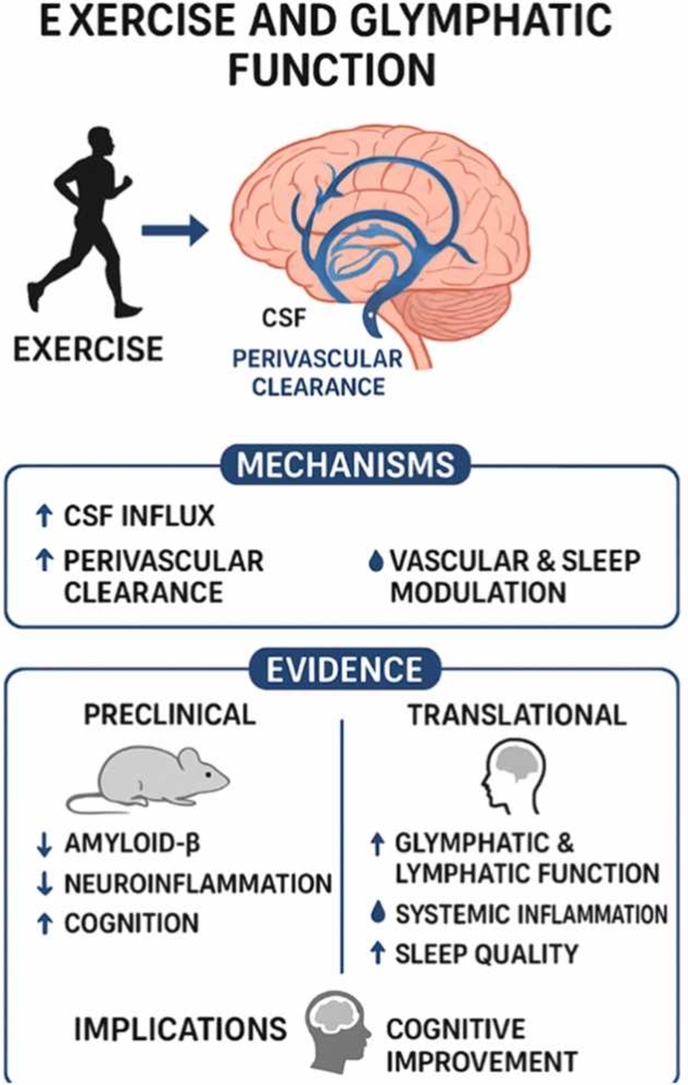
Physical exercise enhances glymphatic function, promoting clearance of neurotoxic proteins like amyloid-β (Aβ) and reducing neuroinflammation. Mechanisms include increased CSF influx, improved perivascular clearance, astrocytic AQP4 polarization, and modulation of vascular and sleep-related processes. Preclinical and human studies show that aerobic and multicomponent exercise improve cognitive performance, vascular health, and sleep quality. Exercise thus represents a non-pharmacological strategy to preserve brain homeostasis and reduce the risk of neurodegenerative diseases, though further longitudinal and multimodal studies are needed.

## 1 Introduction

Unlike most peripheral organs, the central nervous system (CNS) lacks classical lymphatic vessels within its parenchyma (González-Hernández and Mukouyama, 2023, Papadopoulos et al., 2020). Despite this, it must continuously clear metabolic waste to maintain neural homeostasis. The discovery of the glymphatic system in 2012 fundamentally reshaped our understanding of this process (Reddy and van der Werf, 2020, Hablitz and Nedergaard, 2021, Iliff et al., 2012). Through elegant imaging studies using fluorescent tracers and two-photon microscopy, Iliff et al. uncovered a directed movement of cerebrospinal fluid (CSF) into the brain along peri-arterial routes, across the interstitium via astrocytic aquaporin-4 (AQP4) channels, and outward through paravenous pathways (Iliff et al., 2012, Zhu et al., 2023).

Glymphatic function fluctuates with physiological state, reaching its highest activity during slow-wave sleep when interstitial space expansion and vascular pulsatility facilitate CSF flow and waste clearance (Chong et al., 2022; Benveniste,). Impaired glymphatic transport, by contrast, is linked to aging, sleep disturbances, and neurodegeneration, underscoring the system’s sensitivity to lifestyle and physiological factors (Gędek et al., 2023, Chen et al., 2024, Kylkilahti et al., 2021). Among these, physical exercise has emerged as a potent regulator of glymphatic efficiency. Regular aerobic activity enhances vascular pulsatility, arterial compliance, and cerebral blood flow—factors essential for maintaining the pressure gradients that drive CSF movement. Indeed, by improving vascular health, sleep quality, and reducing inflammation, exercise may directly strengthen glymphatic transport and accelerate the clearance of neurotoxic metabolites (Yoo et al., 2025a, Olegário et al., 2024a, Kopeć et al., 2025a, Alnawwar et al., 2023).

Exercise profoundly influences two major regulators of glymphatic function—sleep and inflammation. Regular physical activity enhances sleep quality, increases slow-wave sleep, and stabilizes circadian rhythms, thereby promoting the physiological state in which glymphatic flow and metabolic waste clearance are most active (Reddy and van der Werf, 2020, Alnawwar et al., 2023, Korkutata et al., 2025, Charest and Grandner, 2020, Voumvourakis et al., 2023, Hauglund et al., 2020). In parallel, exercise mitigates neuroinflammation by reducing microglial and astrocytic activation, lowering pro-inflammatory cytokines, and restoring AQP4 polarization on astrocytic endfeet (Hu et al., 2024, Wang et al., 2023, Liu et al., 2022). Together, these effects create an optimal environment for efficient cerebrospinal fluid exchange, facilitating the removal of neurotoxic proteins such as amyloid-β (Aβ) and tau and supporting long-term brain health.

Collectively, current findings indicate that the vascular, sleep-promoting, and anti-inflammatory benefits of exercise translate into measurable enhancements in brain waste clearance in vivo. Exercise thus appears to act not only as a general enhancer of brain health but also as a targeted modulator of glymphatic function, with potential to reduce the risk or progression of neurodegenerative diseases. Despite this promise, research on the exercise–glymphatic relationship remains in its infancy. Further work is needed to elucidate underlying mechanisms, define optimal exercise parameters, and determine whether glymphatic improvements confer lasting protection against cognitive decline. Integrating neuroimaging, molecular biomarkers, and sleep assessment will be crucial to unravel how vascular, circadian, and inflammatory pathways converge.

This review summarizes current evidence on exercise-mediated glymphatic enhancement, explores the underlying mechanisms, and highlights research gaps to guide future investigations on brain health.

## 2 Overview of the anatomy and physiology of the glymphatic system

The glymphatic system is a brain-wide, glia-dependent network that facilitates the clearance of metabolic waste, neurotoxic proteins, and excess interstitial fluid (ISF) from the CNS. It integrates CSF circulation, interstitial fluid dynamics, and astrocytic activity to maintain brain homeostasis and proper neuronal function. (Table 1)

**Table 1:** A clear and well-structured table summarizing the anatomy, physiology, and functional dynamics of the glymphatic system (Jessen et al., 2015, Talbot-Stetsko et al., 2024, Bohr et al., 2022).

Aspect	Description	Key Features / Components
Anatomy	Structural organization of the glymphatic system	• Perivascular spaces (around arteries and veins) • Astrocytes with endfeet lining perivascular spaces • Aquaporin-4 (AQP4) water channels • Interstitial fluid (ISF) pathways • CSF influx via periarterial spaces, efflux via perivenous spaces
Physiology	Mechanisms governing fluid flow and waste clearance	• CSF enters brain along periarterial spaces • Exchange between CSF and ISF facilitated by AQP4 in astrocyte endfeet • Clearance of metabolic waste, neurotoxic proteins (Aβ, tau) • Removal of excess interstitial fluid • Driven by arterial pulsatility and pressure gradients
Functional Dynamics	Modulators and regulation of glymphatic activity	• Sleep (especially slow-wave) expands interstitial space (∼60 %) enhancing CSF–ISF exchange • Physical activity increases arterial pulsatility and CSF flow • Aging and vascular stiffness reduce glymphatic efficiency • Neuroinflammation and astrocyte dysfunction impair clearance • Circadian rhythms and anesthetics can modulate activity

### 2.1 Anatomy of the glymphatic system

CSF enters the brain along periarterial spaces surrounding penetrating arteries, which act as conduits guiding CSF into the ISF of the brain parenchyma. Astrocytic endfeet tightly envelop these perivascular spaces and express high levels of AQP4 water channels, enabling directional CSF–ISF exchange. Interstitial fluid moves through the extracellular space of the parenchyma, carrying metabolic waste and soluble proteins, with flow efficiency dependent on astrocytic coverage, AQP4 polarization, and vascular pulsatility. The system drains toward perivenous spaces and ultimately the meningeal lymphatic vessels, which connect to cervical lymph nodes and the peripheral lymphatic circulation. Cardiac-driven arterial pulsations serve as a major driving force for CSF movement along periarterial spaces, promoting convective flow through the interstitial spaces and enhancing waste clearance (Jessen et al., 2015, Talbot-Stetsko et al., 2024, Bohr et al., 2022). Overall, the glymphatic system is anatomically organized around periarterial CSF inflow, astrocytic AQP4-mediated parenchymal exchange, and perivenous outflow toward meningeal lymphatics, forming an efficient network for brain waste removal.

### 2.2 Physiology and functional dynamics of the glymphatic system

The glymphatic system is a brain-wide clearance network that plays a critical role in maintaining cerebral homeostasis by facilitating the movement of CSF and ISF throughout the brain parenchyma. CSF enters the brain along periarterial spaces that surround penetrating arteries, driven in part by arterial pulsatility, and then exchanges with ISF within the interstitial spaces of neuronal and glial tissue. This exchange is mediated by astrocytic endfeet, which densely express AQP4 water channels, providing low-resistance pathways for fluid transport. The fluid then drains along perivenous spaces, ultimately delivering metabolic waste products, including misfolded proteins such as Aβ and tau, to meningeal lymphatic vessels and the systemic circulation for clearance. This continuous fluid movement not only removes neurotoxic substances but also distributes essential nutrients, signaling molecules, and ions that are necessary for optimal neuronal and glial function, synaptic plasticity, and overall brain metabolism (Jessen et al., 2015, Talbot-Stetsko et al., 2024, Bohr et al., 2022, Mader and Brimberg, 2019).

The efficiency of glymphatic flow is highly dependent on physiological states, vascular dynamics, and external factors that influence cerebrospinal fluid and interstitial fluid movement. Sleep, particularly slow-wave sleep, plays a pivotal role in enhancing glymphatic clearance by expanding the interstitial space by up to 60 %, which reduces resistance to fluid flow and promotes robust CSF–ISF exchange. This expansion is thought to be mediated by astrocytic regulation of extracellular volume and the rhythmic activity of the vasculature during non-rapid eye movement sleep (Reddy and van der Werf, 2020, van Hattem et al., 2024). Enhanced glymphatic activity during sleep facilitates the removal of neurotoxic proteins, including Aβ and tau, and contributes to the consolidation of memory, synaptic remodeling, and cognitive restoration (van Hattem et al., 2024, Mendelsohn and Larrick, 2013, Lyckenvik et al., 2025).

Of note, the results reported by Xie et al. (2013) (Xie et al., 2013) and Miao et al. (2024) (Miao et al., 2024) presented fundamentally different interpretations of how sleep influences waste clearance from the brain. Xie and colleagues, using in vivo two-photon imaging and radiolabeled tracers, demonstrated that sleep and anesthesia markedly enhance CSF influx and promote the clearance of Aβ. They proposed that during sleep, expansion of the interstitial space facilitates convective exchange between CSF and interstitial fluid via the glymphatic pathway, and that this process represents a major restorative function of sleep (Xie et al., 2013). In contrast, Miao et al., (Miao et al., 2024) using fluorescence photometry and histological analyses, observed that both sleep and anesthesia reduce the overall clearance of fluorescent tracers from the brain, while diffusion coefficients remain unchanged between arousal states (Miao et al., 2024). These findings suggest that solute transport in the brain is primarily diffusion-driven rather than convective, thereby challenging the central premise of the glymphatic hypothesis.

Several factors may account for the divergence between these two studies. (Xie et al., 2013). focused on CSF influx into the parenchyma, whereas (Miao et al., 2024). measured overall efflux and tracer clearance from the brain; thus, they examined different phases of the fluid transport process. Differences in tracer characteristics—such as molecular size, charge, and binding properties—may also affect the apparent rate and mechanism of solute movement. Furthermore, the type and depth of anesthesia used could substantially alter vascular pulsatility, intracranial pressure, and CSF dynamics; for instance, ketamine–xylazine used by Xie et al (Xie et al., 2013). mimics natural slow-wave sleep with preserved arterial pulsation, while dexmedetomidine or pentobarbital applied by Miao et al (Miao et al., 2024). suppress cardiovascular activity and may thereby diminish CSF flow. Methodological differences in imaging techniques, timing of tracer measurement, and signal quantification could further contribute to the inconsistent outcomes. Biological variability, including animal strain, age, and astrocytic AQP4 expression, may also influence the efficiency of CSF–interstitial fluid exchange (Xie et al., 2013, Miao et al., 2024). Collectively, these studies underscore the complexity of brain fluid dynamics and indicate that both convective and diffusive processes may operate under different physiological conditions. The apparent contradiction between Xie et al. and Miao et al. likely reflects methodological and physiological heterogeneity rather than a fundamental incompatibility (Xie et al., 2013, Miao et al., 2024). Further research employing standardized imaging protocols and precise physiological control is needed to clarify how sleep, anesthesia, and vascular function interact to regulate waste clearance from the brain.

Notably, recent studies have highlighted the crucial role of α-syntrophin (Snta1), a key scaffolding protein, in maintaining AQP4 polarization at astrocytic endfeet surrounding cerebral blood vessels (Amiry-Moghaddam et al., 2003, Bragg et al., 2006, Rao et al., 2021). In Snta1 knockout (KO) mice, AQP4 becomes mislocalized, leading to depolarization around the vasculature and a marked reduction in glymphatic clearance of interstitial solutes. This finding emphasizes that proper AQP4 localization is essential for efficient cerebrospinal fluid–interstitial fluid exchange and suggests that disruptions in astrocytic scaffolding can significantly impair brain waste clearance, potentially contributing to neurodegenerative processes (Pedersen et al., 2023, Mestre et al., 2018a).

Physical activity further modulates glymphatic dynamics by increasing arterial pulsatility, which acts as a mechanical driving force for CSF movement along periarterial pathways, and by improving the polarization and expression of AQP4 channels on astrocytic endfeet, thereby optimizing perivascular fluid transport. Exercise-induced improvements in vascular compliance and cerebral blood flow enhance metabolic support to neurons and glial cells, while simultaneously promoting the clearance of inflammatory mediators and oxidative byproducts (Olegário et al., 2024a, Xie et al., 2024, Attwell et al., 2010). In contrast, aging, traumatic brain injury, and neurodegenerative diseases such as Alzheimer’s and Parkinson’s impair glymphatic transport through multiple mechanisms, including reduced arterial pulsatility, loss of AQP4 polarization, thickening of basement membranes, and decreased interstitial fluid exchange. These impairments lead to the accumulation of metabolic waste, chronic neuroinflammation, synaptic dysfunction, and ultimately progressive cognitive decline, illustrating how disruptions in glymphatic function can exacerbate both structural and functional deficits in the aging or diseased brain (Buccellato et al., 2022, Jia et al., 2025, Sun et al., 2025a).

The glymphatic system’s neuroprotective role extends beyond waste clearance. Indeed, by efficiently removing metabolic byproducts, inflammatory mediators, and aggregated proteins, it preserves neuronal integrity, reduces oxidative stress, and supports synaptic plasticity. The system also contributes to maintaining the ionic and osmotic balance in the brain’s extracellular space, which is essential for proper neuronal signaling and network activity (Krings et al., 2025, Gu et al., 2022, Kopeć et al., 2025b). Impaired glymphatic function has been associated not only with classical neurodegenerative conditions but also with psychiatric and neuroinflammatory disorders, suggesting that its proper functioning is critical for both cognitive and emotional health. Thus, the glymphatic system represents a fundamental component of brain physiology, linking cerebrospinal fluid dynamics, astrocyte function, vascular pulsatility, and metabolic homeostasis, and highlighting potential therapeutic targets to enhance brain resilience, prevent proteinopathies, and promote cognitive longevity (Krings et al., 2025, Gu et al., 2022, Kopeć et al., 2025b, Zou et al., 2024, Barlattani et al., 2024).

### 2.3 Glymphatic vs. meningeal lymphatic systems

Although both the glymphatic and meningeal lymphatic systems are essential for brain waste clearance, their anatomical and functional distinctions merit clearer delineation. The glymphatic system mediates peri-arterial CSF influx and interstitial solute exchange within the brain parenchyma, driven primarily by arterial pulsatility, endothelial shear stress, and perivascular pressure gradients. Astrocytic AQP4 channels, densely localized at perivascular endfeet, facilitate convective transport of CSF into the interstitial space, enabling efficient clearance of metabolic waste products, including Aβ, tau, and other neurotoxic proteins. Glymphatic transport is modulated by sleep-wake cycles, with slow-wave sleep enhancing interstitial space volume and reducing resistance to fluid flow, as well as by vascular factors such as arterial compliance and capillary density (Jessen et al., 2015, Talbot-Stetsko et al., 2024, Bohr et al., 2022, Mader and Brimberg, 2019, Li et al., 2022).

In contrast, the meningeal lymphatic system provides a downstream, long-range drainage pathway, conveying CSF-derived solutes from the subarachnoid space and perivascular compartments into deep cervical lymph nodes and ultimately the systemic circulation (Feng et al., 2025, Hershenhouse et al., 2019). These vessels are anatomically distinct, lined by endothelial cells expressing lymphatic markers such as LYVE-1, PROX1, and podoplanin, and equipped with valves that ensure unidirectional flow. Their function is influenced by intracranial pressure, vascular pulsatility, systemic inflammation, and endothelial integrity. Disruption of meningeal lymphatic patency has been shown to impair the clearance of amyloid and other macromolecules, exacerbating neuroinflammatory and neurodegenerative processes (Hershenhouse et al., 2019, Zhang et al., 2025, Rego et al., 2023).

Although interconnected, the glymphatic and meningeal lymphatic systems operate through complementary mechanisms: the glymphatic system ensures local convective transport and interstitial solute exchange, while the meningeal lymphatics provide systemic drainage, completing the circuit for metabolic waste clearance (Licastro et al., 2024, Tian et al., 2022). Understanding these distinctions is critical for interpreting neuroimaging studies and for designing interventions—such as exercise, sleep modulation, or vascular-targeted therapies—aimed at enhancing brain clearance. Inclusion of a concise figure contrasting peri-arterial glymphatic flow with meningeal lymphatic drainage would greatly improve conceptual clarity, particularly for clinicians and imaging researchers, by visually linking anatomical structures with their functional roles in CNS homeostasis.

### 2.4 MRI-based assessment of glymphatic function

Non-invasive neuroimaging techniques have become indispensable tools for studying glymphatic function in both preclinical and human studies, allowing detailed characterization of fluid transport dynamics in the CNS.

Diffusion Tensor Imaging–Analysis along the Perivascular Space (DTI-ALPS) is a widely utilized MRI-based technique for the indirect assessment of glymphatic function. This method exploits the anisotropic diffusion of water molecules along perivascular spaces, particularly those aligned with medullary veins, to estimate the efficiency of CSF–ISF exchange (Taoka et al., 2024). Indeed, by calculating water diffusivity along specific axes corresponding to perivascular orientations, DTI-ALPS provides a quantitative proxy of glymphatic activity. Alterations in ALPS indices have been linked to aging, cerebral small vessel disease, and the accumulation of neurotoxic proteins such as Aβ and tau, reflecting impaired clearance along perivascular pathways (Ai et al., 2025, Clark et al., 2024). Beyond fluid dynamics, DTI-ALPS captures subtle microstructural changes in surrounding white matter and perivascular architecture, allowing researchers to correlate fluid transport efficiency with tissue integrity and structural connectivity. Importantly, this technique can detect early glymphatic dysfunction even before overt clinical symptoms emerge, making it a valuable tool for translational studies in neurodegeneration (Taoka et al., 2024, Clark et al., 2024, Yu et al., 2025). Recent large-scale population evidence further supports the robustness—but modest magnitude—of associations between diffusion-derived glymphatic markers and demographic, sleep, and cognitive factors. In a UK Biobank analysis of 17,723 participants, Clark et al. demonstrated that the DTI-ALPS index is consistently associated with age, sex, longest uninterrupted sleep window, and cognitive performance, replicating patterns previously reported in smaller cohorts. Notably, however, effect sizes were substantially attenuated at the population level, underscoring considerable inter-individual variability and highlighting the importance of cautious interpretation when inferring glymphatic function from ALPS-derived metrics. These findings position DTI-ALPS as a stable but relatively insensitive marker, reinforcing the need for complementary physiological and multimodal validation when linking glymphatic activity to clinical or cognitive outcomes (Clark et al., 2024). In parallel, emerging large-scale genetic evidence further substantiates the biological relevance of diffusion-derived glymphatic markers while refining their interpretive scope. A recent genome-wide association study in over 31,000 UK Biobank participants identified 17 genome-wide significant loci and 161 candidate genes associated with ALPS indices, with partial replication across independent cohorts. Notably, shared genetic architecture was observed between the ALPS index, ventricular volume, and CSF tau levels, implicating loci such as *GMNC* and *C16orf95* as convergent substrates linking glymphatic activity, neurodegeneration, and brain structural integrity. Together with population-level phenotypic analyses showing robust yet modest effect sizes, these findings position the DTI-ALPS metric as a stable, genetically informed marker whose sensitivity to glymphatic clearance likely reflects complex, multilevel influences rather than a direct proxy of glymphatic function per se (Huang et al., 2025).

Dynamic T1 mapping complements structural diffusion-based assessments by providing functional information on CSF–ISF exchange. This approach tracks the temporal distribution and kinetics of MRI contrast agents, such as gadolinium-based tracers, within the CSF and brain parenchyma (Solé-Guardia et al., 2025, Ringstad et al., 2023). By monitoring the rate of tracer penetration into perivascular and interstitial compartments, dynamic T1 mapping allows direct estimation of CSF influx and regional variations in glymphatic activity. Areas with delayed contrast accumulation indicate impaired or slowed CSF–ISF exchange, providing mechanistic insights into factors that modulate glymphatic flow, including sleep state, vascular pulsatility, and physical activity. Moreover, this technique enables correlation of functional glymphatic metrics with cognitive performance or disease progression in both preclinical and human studies (Lee et al., 2021).

4D flow MRI represents a more recent innovation for investigating glymphatic physiology by providing high-resolution, time-resolved quantification of CSF movement throughout the ventricles, subarachnoid space, and perivascular channels. This method measures velocity vectors of CSF synchronized with cardiac and respiratory cycles, enabling assessment of flow directionality, pulsatility, and convective transport, which are essential drivers of glymphatic clearance. Alterations in 4D flow metrics, such as reduced peak velocity, diminished pulsatility, or irregular flow patterns, have been associated with arterial stiffening, cerebrovascular disease, and sleep disturbances, providing critical insight into the interplay between vascular function, physiology, and brain waste clearance. Thus, by integrating these measurements with structural and functional imaging, 4D flow MRI offers a comprehensive framework for understanding the mechanisms underlying glymphatic dysfunction and for evaluating interventions aimed at enhancing brain clearance (Rivera-Rivera et al., 2024, Kaur et al., 2020, Șerban et al., 2025).

Physiologically, these imaging methods serve as surrogate markers of CSF–ISF exchange rates and perivascular transport efficiency. For instance, a reduction in ALPS index or abnormal 4D flow dynamics indicates slower convective flow along perivascular spaces, potentially leading to the accumulation of neurotoxic metabolites (Hou et al., 2025, Firbank et al., 2025). Dynamic T1 mapping further informs on solute transport kinetics, enabling quantification of regional heterogeneity in glymphatic clearance (Benveniste et al., 2020, Xue et al., 2020). Together, these techniques provide a multidimensional view of the system, linking structural and functional aspects of fluid transport.

Emerging imaging modalities are further enhancing the translational relevance of glymphatic research. Novel PET tracers, including radiolabeled compounds designed to follow CSF flow or bind specifically to perivascular compartments, offer quantitative assessment of glymphatic function in vivo. These tracers enable direct evaluation of clearance efficiency in humans and the monitoring of disease progression or therapeutic intervention (Peltoniemi et al., 2025). In parallel, ultra-fast MRI sequences, such as echo-planar imaging with high temporal resolution, allow real-time visualization of perivascular fluid motion, capturing rapid pulsatile flow patterns that are tightly coupled to cardiac and respiratory cycles. These advanced imaging approaches complement traditional MRI techniques, enabling a more comprehensive characterization of glymphatic dynamics (Kiviniemi et al., 2025, Kiviniemi et al., 2015).

## 3 Evidence from the role of exercise in glymphatic clearance

Research on the impact of exercise on glymphatic clearance is still limited. Both preclinical and emerging clinical evidence indicate that physical exercise enhances glymphatic clearance. In animal models, voluntary running or aerobic exercise increases CSF flow, restores AQP4 polarization, reduces Aβ accumulation, and improves cognitive performance, with effects being most pronounced when interventions are initiated early or when AQP4 function is intact. In humans, structured aerobic or combined exercise programs are associated with increased glymphatic and meningeal lymphatic function, improved vascular dynamics, reduced systemic inflammation, and enhanced sleep quality, suggesting that physical activity may support brain waste clearance and cognitive health through multiple complementary mechanisms. (Table 2)

**Table 2:** Exercise-Induced Enhancement of Glymphatic Clearance: Evidence and Mechanisms.

Study Category	Experimental Model / Population	Exercise Modality	Exposure (Duration & Intensity)	Glymphatic / Lymphatic Outcomes	Neurobiological Effects	Cognitive / Functional Outcomes	Key Mechanistic Interpretation		Ref
Clinical									
	Healthy adults	Cycling	12 weeks, moderate intensity	↑ Glymphatic influx (T1 mapping); ↑ meningeal lymphatic flow	↓ Plasma inflammatory markers (S100A8, S100A9, PSMA3, DEFA1A3)	Improved sleep quality and general well-being	Enhanced arterial pulsatility; systemic anti-inflammatory effects; sleep-linked facilitation of glymphatic transport		(Yoo et al., 2025b)
	MCI or AD	Multicomponent aerobic + resistance training	12 weeks	↑ Expected glymphatic and meningeal lymphatic activity; ↑ cerebral perfusion	Improved vascular and metabolic brain milieu	↑ Cognitive and functional performance	Convergent vascular–glymphatic–sleep interactions supported by biomarker changes		(Olegário et al., 2024b)
Preclinical									
	APP/PS1 mice (early vs. late intervention; Aqp4 −/− subset)	Voluntary wheel running	2 months (onset at 3 or 7 months); knockout comparison	Early: restored CSF–ISF exchange; Late: impaired exchange; KO: severe dysfunction	↓ Aβ burden, ↓ glial reactivity (early only); restored AQP4 polarity	Early: memory improvement; Late: minimal benefit; KO: persistent decline	AQP4-dependent glymphatic enhancement; timing of exercise is critical		(Liu et al., 2022)
	Aged mice	Voluntary wheel running	5 weeks	↑ CSF tracer influx	↓ Astrocyte and microglial activation; ↓ Aβ; ↑ synaptic proteins (PSD95)	↑ Spatial learning and memory	Upregulation and polarization of AQP4; reduced neuroinflammation improves convective flow		(He et al., 2017)
	Young C57BL/6 J female mice	Voluntary wheel running (∼6 km/day)	5 weeks	↑ CSF influx in hypothalamus, ventral cortex, MCA territories	Preserved neuronal homeostasis	Maintained cognitive performance	Exercise enables glymphatic function even during wakefulness		(von Holstein-Rathlou et al., 2018)
	APP/PS1 mice	Aerobic swimming ± AQP4 inhibition	4 weeks	↑ CSF clearance (abolished with AQP4 inhibition)	↓ Neuroinflammation	↑ Spatial learning and memory (blocked by AQP4 inhibition)	Direct confirmation of AQP4-mediated exercise effects on glymphatic clearance		(Liang et al., 2025a)

### 3.1 Clinical evidence on exercise-induced enhancement of glymphatic function

Recent clinical investigations have begun to translate the compelling preclinical findings into human contexts, demonstrating that regular physical exercise can modulate cerebrospinal and lymphatic fluid dynamics, enhance vascular and neuroimmune homeostasis, and ultimately support cognitive health. These studies collectively emphasize the multidimensional nature of exercise-induced benefits, encompassing cerebrovascular, glymphatic, and systemic mechanisms.

In healthy adults, a 12-week program of moderate-intensity cycling resulted in increased glymphatic CSF influx, quantified using T1-mapping MRI, alongside augmented meningeal lymphatic flow. These central effects were paralleled by significant reductions in circulating inflammatory proteins, including S100A8, S100A9, PSMA3, and DEFA1A3. Participants also reported improvements in general well-being and sleep quality, implicating exercise-induced increases in vascular pulsatility, systemic anti-inflammatory signaling, and sleep-dependent glymphatic facilitation as key mediators of these benefits. These proteins are known mediators of oxidative stress, leukocyte activation, and neuroinflammation; thus, their suppression indicates a shift toward an anti-inflammatory systemic state conducive to cerebrovascular health (Yoo et al., 2025b).

Additionally, an increase in J-chain—a component associated with immunoglobulin polymerization and mucosal immunity—may reflect adaptive immune regulation in response to regular exercise. Participants also reported subjective improvements in sleep quality and overall well-being, both of which are physiologically relevant since deep sleep is known to substantially enhance glymphatic transport. The convergence of these findings suggests that aerobic exercise augments glymphatic function by improving vascular pulsatility, optimizing CSF–ISF exchange, dampening inflammation, and reinforcing sleep-dependent clearance mechanisms (Yoo et al., 2025b).

Parallel evidence has emerged in patients with mild cognitive impairment (MCI) or early AD, conditions characterized by early glymphatic dysfunction and impaired waste clearance. In a 12-week multicomponent exercise program combining aerobic and resistance training, participants showed measurable increases in expected glymphatic and meningeal lymphatic function, accompanied by improved cerebral perfusion as indicated by neuroimaging data. Enhanced blood flow and vascular compliance likely facilitate perivascular fluid movement, a key driver of glymphatic activity. Clinically, these physiological improvements translated into enhanced cognitive and functional performance, including better memory retention and daily living capabilities. Mechanistically, these effects appear to arise from integrated vascular, glymphatic, and sleep-mediated pathways. Exercise promotes vasomotor pulsatility and endothelial nitric oxide availability, which drive perivascular CSF flow; it simultaneously reduces systemic and neuroinflammatory burden, preserving astrocytic AQP4 polarization and perivascular integrity. Moreover, regular exercise contributes to improved sleep architecture—particularly the duration of slow-wave sleep—which synergistically enhances glymphatic clearance. Collectively, these adaptations foster a neuroprotective milieu that supports the removal of neurotoxic proteins such as Aβ and tau, stabilizes neural connectivity, and improves cognitive resilience (Olegário et al., 2024b).

Overall, accumulating clinical evidence supports the notion that exercise acts as a non-pharmacological enhancer of glymphatic and meningeal lymphatic function, with downstream benefits for brain homeostasis and cognition. Thus, by integrating vascular regulation, immune modulation, and sleep physiology, physical activity represents a holistic intervention strategy that targets the multiple converging mechanisms implicated in neurodegenerative and cognitive disorders.

### 3.2 Preclinical evidence on exercise-induced enhancement of glymphatic function

A series of preclinical investigations have demonstrated that physical exercise exerts a profound modulatory effect on glymphatic transport, amyloid clearance, and cognitive performance across various mouse models of aging and Alzheimer’s disease (AD).

In APP/PS1 transgenic mice, voluntary wheel running elicited markedly different outcomes depending on the timing of intervention and the presence of AQP4. When exercise was initiated at an early disease stage (3 months of age), mice exhibited reduced Aβ deposition, attenuated glial reactivity, restored AQP4 polarity, and enhanced glymphatic clearance, accompanied by improved cognitive performance. In contrast, late-stage intervention (7 months of age) failed to produce comparable benefits, as AQP4 depolarization persisted, CSF–ISF exchange remained impaired, and cognitive gains were minimal. Moreover, Aqp4 knockout mice displayed severe glymphatic dysfunction, elevated amyloid accumulation, and sustained cognitive decline, underscoring the critical role of AQP4 in mediating the exercise-induced enhancement of waste clearance and neural function. Collectively, these findings highlight that timing of intervention and AQP4 integrity are essential determinants of the neuroprotective effects of physical activity (Liu et al., 2022).

Complementary evidence from aged wild-type mice revealed that five weeks of voluntary wheel running significantly increased CSF tracer movement, reduced astrocytic and microglial activation, and decreased Aβ accumulation. These physiological changes were paralleled by improvements in spatial learning and memory, along with elevated expression and polarization of AQP4 and enhanced convective CSF–ISF flow, suggesting a dual role of exercise in promoting glymphatic efficiency and mitigating neuroinflammation (He et al., 2017).

Further, studies in young awake C57BL/6 J female mice demonstrated that sustained voluntary running (∼6 km/day) increased CSF influx particularly in the hypothalamus, ventral cortex, and middle cerebral artery territory. The observation that these effects occurred even during wakefulness suggests that exercise augments brain waste clearance independently of sleep states, thereby contributing to preserved cognitive performance (von Holstein-Rathlou et al., 2018). Finally, investigations using APP/PS1 mice subjected to aerobic swimming provided additional mechanistic confirmation of AQP4 involvement. Four weeks of swimming exercise enhanced CSF clearance and reduced neuroinflammatory responses, while co-administration of an AQP4 inhibitor abolished both physiological and cognitive benefits. This strongly supports the notion that exercise-induced cognitive enhancement is contingent upon AQP4-dependent glymphatic activity (Liang et al., 2025a).

Together, these preclinical studies converge on a consistent theme: regular physical exercise facilitates glymphatic clearance, attenuates neuroinflammation, and enhances cognitive outcomes, primarily through mechanisms involving AQP4 expression and polarization, optimized CSF–ISF exchange, and preserved vascular-glial communication.

### 3.3 Translational relevance: linking glymphatic mechanisms to clinical outcomes

While the glymphatic system’s physiological mechanisms—such as perivascular CSF–ISF exchange, astrocytic AQP4 polarization, and pulsatile vascular flow—are increasingly well characterized, their clinical implications remain a critical area of translation. Impairments in glymphatic clearance have been directly associated with cognitive decline, small-vessel disease (SVD), and the radiological burden of enlarged perivascular spaces (EPVS) (Ai et al., 2025, Zhang et al., 2021). EPVS, which can be quantified using high-resolution MRI, are considered surrogate markers of impaired interstitial fluid drainage and correlate with aging, cerebrovascular dysfunction, and early stages of neurodegenerative disorders such as AD (Ramirez et al., 2016).

Reduced glymphatic efficiency has also been linked to declines in memory, executive function, and global cognition, highlighting the functional consequences of disrupted waste clearance (Bao et al., 2025, Chong et al., 2025). Notably, glymphatic-based imaging biomarkers provide a translational bridge between mechanism and outcome. (Table 3)

**Table 3:** Glymphatic Imaging Biomarkers: Mechanisms, Clinical Relevance, and Therapeutic Potential.

Imaging Methods	Measure	Clinical Relevance	Therapeutic Potential	Ref
DTI-ALPS	Perivascular water diffusion; CSF–ISF exchange	Low ALPS → impaired glymphatic function, cognitive decline, SVD	Monitor effects of exercise, sleep, or vascular therapies	(Taoka et al., 2017, Wang et al., 2025)
Dynamic T1 / DCE-MRI	CSF influx and solute clearance	Slowed CSF flow → early cognitive impairment	Track improvements in glymphatic transport	(Koundal et al., 2024, Benveniste et al., 2021, Ayyappan et al., 2026)
Free-Water Imaging	Extracellular fluid fraction	High free water → EPVS, SVD, neuroinflammation	Assess reduction in interstitial fluid stagnation	(Duering et al., 2018, Sun et al., 2024)
4D Flow MRI	CSF velocity and pulsatility	Altered flow → vascular dysfunction, reduced clearance	Evaluate changes in perivascular flow after interventions	(Rivera-Rivera et al., 2024, Vikner et al., 2024)
EPVS Quantification	Perivascular space dilation	EPVS burden → SVD, cognitive decline	Monitor progression or stabilization with therapy	(Bown et al., 2023, Chen et al., 2025a)
Emerging PET / Ultra-Fast MRI	Direct perivascular flow imaging	Early detection of glymphatic impairment	Assess rapid response to interventions	(Botta et al., 2025, Zhou et al., 2025)

These imaging biomarkers are highly relevant for early diagnosis and risk stratification. For example, reductions in ALPS indices may indicate impaired perivascular water diffusion and compromised CSF–ISF exchange (Taoka et al., 2024), while slowed CSF influx rates measured by dynamic T1 mapping or DCE-MRI reflect decreased glymphatic clearance capacity (Bohr et al., 2022, Benveniste et al., 2021). Similarly, an increased burden of EPVS, detectable on high-resolution MRI, is associated with interstitial fluid stagnation, microvascular dysfunction, and early neurodegenerative changes (Bown et al., 2022). Collectively, these metrics can identify individuals at elevated risk for cognitive impairment, AD, or small-vessel cerebrovascular pathology before overt clinical symptoms manifest.

Beyond diagnosis, these biomarkers provide a quantitative framework for therapeutic monitoring. Interventions such as aerobic or resistance exercise, sleep optimization, and vascular-targeted therapies can be evaluated for their ability to enhance CSF–ISF exchange, restore perivascular fluid dynamics, and reduce accumulation of neurotoxic metabolites such as Aβ and tau (Lyckenvik et al., 2025, Liang et al., 2025b). Imaging-derived metrics can be correlated with functional outcomes, including performance on cognitive batteries, neuropsychological assessments, and longitudinal measures of structural integrity or EPVS progression. This approach enables clinicians and researchers to quantify treatment efficacy, determine dose- or duration-dependent effects, and mechanistically link interventions to improvements in vascular, glymphatic, and cognitive health. Importantly, combining these imaging biomarkers with physiological and behavioral measures allows for multimodal evaluation, providing a more comprehensive understanding of how therapeutic strategies modulate brain clearance pathways and contribute to neuroprotection (Roalf et al., 2014, Chen et al., 2025a, Chen et al., 2025b).

In summary, connecting glymphatic mechanisms to clinically measurable outcomes not only deepens our understanding of brain clearance physiology but also establishes glymphatic imaging as a powerful tool for early detection, risk stratification, and evaluation of therapeutic strategies in neurodegenerative and cerebrovascular disorders.

#### 3.3.1 Inflammation, blood-brain barrier (BBB), and glymphatic dysfunction

Systemic inflammation critically modulates glymphatic function through its impact on the blood-brain barrier (BBB) and astrocytic regulation. Elevated peripheral pro-inflammatory cytokines, including tumor necrosis factor-alpha (TNF-α), interleukin-1 beta (IL-1β), and interleukin-6 (IL-6), induce endothelial activation, disrupt tight junction proteins such as claudin-5 and occludin, and increase BBB permeability (Gryka-Marton et al., 2025). This endothelial dysfunction alters perivascular homeostasis and impairs astrocytic AQP4 polarization at endfeet, thereby reducing the efficiency of CSF–ISF exchange. The resulting slowdown in glymphatic clearance diminishes the removal of neurotoxic proteins, including Aβ and tau, contributing to their accumulation in the brain parenchyma (Zou et al., 2024, Yue et al., 2024, Rani et al., 2023).

Chronic systemic inflammation also affects meningeal lymphatic vessels, reducing their patency and downstream drainage capacity. Molecularly, endothelial activation and oxidative stress elevate reactive oxygen species (ROS) and matrix metalloproteinase (MMP) activity, which can degrade extracellular matrix components surrounding lymphatic vessels, further impeding solute transport (Kar et al., 2010). Perivascular astrocytes respond to inflammatory signaling through activation of NF-κB and MAPK pathways (Linnerbauer et al., 2020), which can modify cytoskeletal organization and impair astrocyte–vascular coupling (Qin et al., 2024). Together, these effects create a feed-forward loop where systemic inflammation, vascular dysfunction, and impaired glymphatic clearance exacerbate neuroinflammation and cognitive decline.

This interplay is particularly relevant in age-related, metabolic, and neurodegenerative disorders, such as AD, diabetes, and hypertension, where chronic low-grade systemic inflammation, endothelial stiffening, oxidative stress, and dysregulated glucose metabolism converge to impair vascular compliance, pericyte function, and astrocytic AQP4 polarization, collectively compromising glymphatic clearance. In AD, for example, elevated circulating cytokines and ROS disrupt tight junction proteins in the BBB, reduce meningeal lymphatic vessel patency, and slow CSF–ISF exchange, promoting the accumulation of Aβ and tau (Kim et al., 2024, Yang et al., 2022).

Evidence from preclinical and clinical studies suggests that interventions targeting systemic inflammation and vascular health can restore glymphatic efficiency. Regular aerobic exercise promotes endothelial nitric oxide production, enhances arterial pulsatility, stimulates angiogenesis, and supports AQP4 polarization at astrocytic endfeet, thereby improving CSF–ISF exchange and perivascular drainage (Liang et al., 2021, Green et al., 2004, Königstein et al., 2023). Thus, by integrating systemic inflammatory status, BBB integrity, astrocytic function, vascular dynamics, and glymphatic efficiency, this framework provides a mechanistic rationale for early interventions in neurodegenerative and cerebrovascular diseases, highlighting the translational potential of therapies that target peripheral and central inflammatory pathways to preserve brain clearance and cognitive health.

## 4 Vascular–astrocytic interactions in glymphatic function

Astrocytic endfeet, enriched with AQP4 water channels, are central to glymphatic function, directing CSF into the interstitial space and facilitating CSF–ISF exchange along perivascular pathways. However, efficient glymphatic transport depends not only on astrocytic regulation but also on vascular dynamics, establishing a tightly coupled, bidirectional system in which cerebrovascular properties influence astrocytic function and, conversely, astrocytes modulate vascular tone (Hablitz and Nedergaard, 2021, Sims et al., 2025, Vittani et al., 2025).

Exercise-induced vascular adaptations provide a critical mechanism for enhancing the coupling between cerebrovascular dynamics and astrocytic regulation of glymphatic function. Regular aerobic activity stimulates angiogenesis through upregulation of vascular endothelial growth factor (VEGF) and related pro-angiogenic factors, leading to increased capillary density and expanded microvascular surface area (Jiang et al., 2025, Ross et al., 2023, Karakilic et al., 2021). These structural changes improve regional tissue perfusion and create wider, more compliant perivascular spaces that facilitate convective CSF–ISF exchange. Simultaneously, elevated endothelial shear stress resulting from increased blood flow activates endothelial nitric oxide synthase (eNOS), increasing the production of nitric oxide, a potent vasodilator, as well as prostacyclin (PGI₂), which together enhance arterial compliance and pulsatility. These pulsatile forces act as the primary driving mechanism for CSF movement along perivascular pathways, generating directional convective flow that efficiently transports interstitial solutes toward meningeal lymphatic vessels and downstream systemic clearance pathways (Davis et al., 2001, Sriram et al., 2016, Di Francescomarino et al., 2009).

At the cellular level, endothelial cells respond to shear stress by activating mechanotransduction pathways, including PI3K/Akt and MAPK/ERK signaling, which further support endothelial health, vascular remodeling, and maintenance of barrier integrity. These vascular adaptations also modulate pericyte contractility via calcium- and RhoA/ROCK-dependent pathways, ensuring that capillary diameter and perivascular space patency are optimized for CSF–ISF transport (Chatterjee, 2018, Di et al., 2023, Gathings et al., 2024). Therefore, by enhancing perfusion, vascular compliance, and perivascular flow dynamics, exercise establishes a physiologically favorable environment that directly reinforces astrocytic AQP4 polarization, gliovascular signaling, and overall glymphatic efficiency, thereby supporting the clearance of metabolic waste and neurotoxic proteins such as Aβ and tau.

Pericytes, contractile cells surrounding capillaries, play a crucial role in modulating glymphatic flux by regulating microvascular tone, capillary diameter, and the patency of perivascular spaces. Exercise and improved cerebral perfusion enhance pericyte-mediated relaxation through calcium-dependent signaling and RhoA/ROCK pathways, which dilate capillaries and expand perivascular spaces, thereby increasing the efficiency of CSF–ISF exchange (Whalley, 2024, Andrade-Guerrero et al., 2023, Sun et al., 2025b). Conversely, pericyte dysfunction, as often observed in aging, hypertension, or cerebrovascular disease, leads to reduced vascular compliance, constricted perivascular spaces, and impaired glymphatic clearance, highlighting their essential role in maintaining effective brain waste removal and interstitial homeostasis (Lin et al., 2024, Whitehead et al., 2023, Cao et al., 2025, Zheng et al., 2020).

Vascular and astrocytic mechanisms are tightly integrated through gliovascular signaling, creating a bidirectional communication network. Astrocytes detect changes in blood flow, shear stress, and oxygen delivery via mechanosensitive channels (e.g., Piezo1) and neurotransmitter receptors, responding by releasing vasoactive molecules including arachidonic acid metabolites, potassium ions (K⁺), prostaglandins, and VEGF (Ballesteros-Gomez et al., 2023, Cibelli et al., 2024, MacVicar and Newman, 2015). These factors locally modulate capillary tone and microvascular flow, which in turn amplify arterial pulsatility. Enhanced pulsatile flow drives convective CSF movement, which is efficiently channeled into the interstitial compartment via polarized AQP4 channels on astrocytic endfeet. This creates a dynamic feedback loop, whereby vascular pulsatility, pericyte regulation, and astrocytic water transport work in concert to optimize glymphatic clearance, maintain interstitial homeostasis, and facilitate the removal of neurotoxic proteins such as Aβ and tau (Mestre et al., 2018b, Fan and Gao, 2025, Bojarskaite et al., 2024).

## 5 Current limitations and future directions

Investigating the intricate relationship between physical exercise and glymphatic function remains a significant scientific challenge, primarily due to methodological limitations and a lack of standardized assessment tools. While the concept of exercise-mediated enhancement of glymphatic clearance is increasingly supported by preclinical studies, translating these findings into reliable, reproducible human data demands a more refined methodological framework and integrative research design.

Despite extensive research over the past decade, findings on the glymphatic system remain heterogeneous, with some studies reporting robust perivascular CSF–ISF exchange and others questioning its existence or functional significance. These discrepancies may arise from differences in species, imaging modalities, tracer types, physiological states, and experimental conditions. Addressing these inconsistencies requires carefully controlled, multimodal studies in both preclinical models and humans, including standardized imaging protocols, longitudinal designs, and integration of vascular, astrocytic, and sleep-related factors. Thus, clarifying these uncertainties will be critical for establishing the physiological relevance of glymphatic clearance and its potential as a therapeutic target.

Importantly, AQP4 plays a dual and context-dependent role in brain physiology. Properly polarized AQP4 at astrocytic endfeet is essential for glymphatic function, facilitating the convective flow of cerebrospinal and interstitial fluids and promoting efficient clearance of metabolic waste and neurotoxic proteins (Verkman et al., 2006, Kokkoris et al., 2025, Huang et al., 2019). Conversely, excessive expression or mislocalization of AQP4 can increase water permeability, contributing to cytotoxic or vasogenic edema (Kokkoris et al., 2025, Huang et al., 2019). As mentioned earlier, physical exercise appears to influence this balance by enhancing AQP4 polarization and glymphatic clearance, potentially promoting the beneficial aspects of AQP4 function without triggering edema. Future studies should aim to elucidate the molecular mechanisms through which exercise regulates AQP4 expression and polarization, determine the optimal type, intensity, and duration of exercise for maximizing waste clearance while maintaining brain water homeostasis, and examine how individual factors such as age, sex, and disease state modulate these effects. Understanding these mechanisms could inform strategies to harness AQP4’s protective role while minimizing its potential deleterious consequences, offering a non-pharmacological approach to improving brain health and reducing neurodegenerative risk. Other limitations and recommendations can be shown in the following subheadings.

### 5.1 Imaging limitations and technical confounders

The DTI-ALPS method has emerged as one of the most widely used non-invasive techniques to estimate glymphatic activity. This approach quantifies the diffusivity of water molecules along perivascular tracts and is valued for its accessibility in conventional MRI platforms (Botta et al., 2025, An et al., 2025, Ringstad, 2024). However, despite its promise, DTI-ALPS is inherently constrained by several confounding variables. The technique’s accuracy is highly sensitive to white matter orientation, scanner-specific spatial resolution, signal-to-noise ratios, and proximity to large vessels, all of which can distort diffusion metrics. Moreover, DTI-ALPS captures only one aspect of glymphatic function—the passive diffusion of water—without reflecting the active convective transport of solutes or the clearance of metabolic waste, such as Aβ or tau. Consequently, it provides a limited and indirect proxy for glymphatic efficiency.

Although the DTI-ALPS algorithm has inherent limitations—such as sensitivity to noise, partial volume effects, challenges in distinguishing small perivascular spaces, and reliance on indirect measures of glymphatic flow—its application in numerous clinical studies has provided important preliminary evidence on human glymphatic function. These studies have revealed associations between glymphatic activity and aging, sleep quality, cognitive status, and neurological disorders. However, the results should be interpreted cautiously, as methodological biases may influence absolute values and subtle regional differences. Rather than questioning these findings outright, they should be viewed as indicative trends that guide further research. Future investigations should aim to validate DTI-ALPS outcomes with complementary imaging techniques, including dynamic contrast-enhanced MRI, intrathecal or intravenous tracer studies, and advanced diffusion modeling. Additionally, integrating multimodal approaches and longitudinal designs could improve reproducibility and allow more precise assessment of glymphatic responses to interventions, such as physical exercise, pharmacological modulation, or sleep optimization. Such efforts would clarify the clinical utility of DTI-ALPS while accounting for its technical limitations.

To overcome these constraints, multimodal imaging paradigms should be developed and validated. Integrating DTI-ALPS with contrast-enhanced MRI, dynamic PET tracers, and 4D flow MRI can provide complementary insights into CSF dynamics, vascular pulsatility, and perivascular exchange. For instance, contrast-enhanced MRI can directly visualize CSF inflow and efflux pathways, while 4D flow imaging quantifies the hemodynamic forces that propel fluid movement along perivascular channels. PET imaging, employing tracers labeled for metabolic waste or blood–brain barrier permeability, can elucidate molecular-level clearance mechanisms. However, the absence of standardized acquisition protocols, analysis pipelines, and validation studies continues to hinder the reproducibility of findings.

Recommendations include establishing international consensus protocols for glymphatic imaging that clearly define optimal MRI sequences, spatial resolutions, and standardized reference parameters. It is also essential to develop cross-validation frameworks that directly compare DTI-ALPS with tracer-based imaging methods to enhance interpretability, accuracy, and cross-study comparability. Furthermore, creating harmonized data repositories across research institutions would enable pooled analyses, foster collaborative meta-analytic efforts, and substantially improve the reproducibility and reliability of glymphatic research findings.

### 5.2 Establishing causality between exercise and glymphatic function

A critical research gap lies in demonstrating causal links between physical exercise and glymphatic enhancement in humans. While animal models have shown clear mechanistic relationships—through direct visualization of tracer clearance, AQP4 polarization, and vascular pulsatility—human studies remain largely correlational. Glymphatic activity is modulated by a constellation of interacting variables, including sleep architecture, vascular health, metabolic rate, and circadian rhythm, making it difficult to isolate the specific contribution of exercise.

Future investigations should prioritize longitudinal intervention studies that manipulate exercise parameters (intensity, frequency, and duration) while simultaneously monitoring confounding factors. Utilizing advanced statistical modeling and mediation analysis could help disentangle direct effects of exercise from indirect effects mediated by improvements in sleep or cardiovascular function.

Recommendations for future research include conducting controlled exercise intervention trials that incorporate mechanistic endpoints, such as cerebrospinal fluid flow velocity, AQP4 distribution, or PET-based tracer clearance, to directly assess the effects of physical activity on glymphatic function. Additionally, dose–response analyses should be applied to determine the optimal intensity, duration, and frequency of exercise needed to achieve measurable improvements in brain clearance. Finally, integrating machine learning approaches could help identify predictive patterns that link exercise parameters, sleep quality, and glymphatic dynamics, providing a more precise understanding of how lifestyle interventions modulate brain waste clearance.

### 5.3 Integrating sleep and cardiovascular metrics

Sleep and vascular dynamics represent key physiological mediators of glymphatic function. Glymphatic transport is maximized during slow-wave sleep, when neuronal activity decreases and the interstitial space expand, facilitating convective CSF–interstitial fluid exchange. Exercise has well-established benefits for sleep architecture—particularly in increasing slow-wave duration—but only a few studies have explicitly connected these changes to glymphatic enhancement. Likewise, exercise-induced improvements in vascular pulsatility, arterial compliance, and heart rate variability likely contribute to the driving forces of perivascular flow, yet these mechanisms remain understudied in this context.

Recommendations for advancing this area of research include incorporating polysomnography and EEG-based sleep staging into exercise studies to directly correlate improvements in sleep quality with glymphatic clearance metrics. Studies should also include comprehensive cardiovascular assessments, such as arterial stiffness, cardiac output, and pulse wave velocity, to evaluate how hemodynamic factors influence perivascular fluid movement. Moreover, developing integrated multimodal models that simultaneously examine the interplay between sleep, vascular function, and exercise will provide deeper mechanistic insights into how these factors collectively shape brain clearance efficiency.

### 5.4 Need for longitudinal and translational studies

Most existing investigations employ short-term exercise interventions, typically lasting a few weeks, which may not capture the cumulative adaptations that underlie durable glymphatic remodeling. Chronic exercise is likely required to induce vascular remodeling, astrocytic AQP4 polarization, anti-inflammatory modulation, and metabolic stabilization, all of which enhance long-term clearance efficiency and neuroprotection. Moreover, the ultimate clinical relevance of exercise-induced glymphatic enhancement depends on its capacity to mitigate cognitive decline and reduce the risk of neurodegenerative diseases such as AD.

Recommendations for future research include designing longitudinal studies extending over several months to years that integrate imaging, physiological, and cognitive endpoints to capture both short- and long-term effects of exercise on glymphatic function. It is also essential to incorporate comprehensive cognitive testing batteries, assessing domains such as memory, executive function, and processing speed, to establish functional correlates of enhanced clearance. Promoting translational collaborations that link human neuroimaging findings with mechanistic animal models will ensure that observed biomarkers accurately reflect physiological improvements. Finally, studies should examine age-, sex-, and disease-specific responses, as glymphatic efficiency and its responsiveness to exercise may differ across populations, informing tailored intervention strategies.

### 5.5 Different exercise types

Although accumulating evidence supports the beneficial effects of physical exercise on glymphatic function, several limitations and gaps remain. Preclinical studies indicate that voluntary wheel running or aerobic swimming enhances CSF–ISF exchange, restores AQP4 polarization, reduces Aβ accumulation, and improves cognitive performance in mouse models. These effects are highly dependent on early intervention, exercise duration, and intact AQP4 function. Clinical studies demonstrate that moderate-intensity cycling or multicomponent aerobic plus resistance training can increase glymphatic and meningeal lymphatic activity, improve cerebral perfusion, reduce systemic inflammation, and enhance cognitive outcomes in both healthy adults and patients with mild cognitive impairment. However, there is a lack of systematic investigation comparing exercise modalities (e.g., resistance training, high-intensity interval training, combined programs), as well as insufficient data on optimal intensity, duration, and frequency for maximizing glymphatic clearance. Future studies should focus on (i) directly comparing different types of exercise, (ii) defining dose–response relationships, (iii) assessing long-term effects on brain clearance and cognitive function, and (iv) exploring underlying molecular mechanisms, including AQP4 regulation and polarization. Addressing these gaps will help develop evidence-based exercise guidelines aimed at optimizing glymphatic function, reducing neuroinflammation, and supporting brain health while minimizing potential adverse effects such as edema.

## 6 Conclusion

Evidence from preclinical and clinical studies indicates that physical exercise enhances glymphatic function, promotes brain waste clearance, and supports cognitive health. In animal models, exercise improves CSF influx, reduces neuroinflammation, attenuates Aβ accumulation, and enhances cognition, largely through AQP4-dependent mechanisms. Human studies corroborate these effects, showing increased glymphatic and meningeal lymphatic activity, improved vascular function, reduced systemic inflammation, and better sleep quality, with corresponding cognitive benefits in both healthy adults and individuals with mild cognitive impairment or early AD. Despite these advances, methodological challenges—such as limitations in imaging techniques, difficulty establishing causality, and short-term intervention designs—highlight the need for longitudinal, multimodal studies. Future research should standardize imaging protocols, assess dose–response relationships, integrate cardiovascular and sleep metrics, and explore age-, sex-, and disease-specific responses.

Overall, exercise emerges as a promising non-pharmacological strategy to enhance glymphatic clearance, support neural homeostasis, and reduce the risk of neurodegenerative disease, emphasizing its potential as a targeted lifestyle intervention for brain health.

## Abbreviation

**CNS**: central nervous system; **CSF**: cerebrospinal fluid; **AQP4**: aquaporin-4**; Aβ**: Amyloid-β; **ISF**: interstitial fluid; **MCI**: mild cognitive impairment; **DTI-ALPS**: Diffusion Tensor Imaging–Analysis along the Perivascular Space; **BBB**: blood-brain barrier.

## CRediT authorship contribution statement

**Arman Ghayourvahdat:** Writing – review & editing, Writing – original draft, Investigation, Conceptualization. **Hannaneh Azimizonuzi:** Writing – review & editing, Writing – original draft, Visualization, Investigation. **Moslem Ahmed:** Writing – review & editing, Writing – original draft, Validation, Supervision, Conceptualization.

## Ethical approval and consent to participate

Since this was a review and no one actively participated, it was not essential for this study.

## Authors’ contributions

All authors (A.Gh., H.A., and M.A.) listed have made a substantial, direct, and intellectual contribution to the work, and approved it for publication. All authors read and approved the final manuscript.

## Consent for publication

Not applicable.

## Funding

The study did not receive any specific support from public, commercial, or nonprofit funding organizations.

## Declaration of Competing Interest

There are not any competing interests to declare.

## Data Availability

All is contained in the paper.
